# Investigation of Lithium Ion Diffusion of Graphite Anode by the Galvanostatic Intermittent Titration Technique

**DOI:** 10.3390/ma14164683

**Published:** 2021-08-19

**Authors:** Jong Hyun Park, Hana Yoon, Younghyun Cho, Chung-Yul Yoo

**Affiliations:** 1 Energy Conversion & Storage Materials Research Laboratory, Korea Institute of Energy Research, 152 Gajeong-ro, Yuseong-gu, Daejeon 34129, Korea; whdgus615@gmail.com (J.H.P.); hanayoon@kier.re.kr (H.Y.); 2 Department of Energy Systems Engineering, Soonchunhyang University, Asan 31538, Korea; yhcho@sch.ac.kr; 3 Department of Chemistry, Mokpo National University, Muan-gun 58554, Korea

**Keywords:** graphite, diffusion coefficient, galvanostatic intermittent titration technique, quasi-equilibrium open circuit potential, lithium-ion battery

## Abstract

Graphite is used as a state-of-the-art anode in commercial lithium-ion batteries (LIBs) due to its highly reversible lithium-ion storage capability and low electrode potential. However, graphite anodes exhibit sluggish diffusion kinetics for lithium-ion intercalation/deintercalation, thus limiting the rate capability of commercial LIBs. In order to determine the lithium-ion diffusion coefficient of commercial graphite anodes, we employed a galvanostatic intermittent titration technique (GITT) to quantify the quasi-equilibrium open circuit potential and diffusion coefficient as a function of lithium-ion concentration and potential for a commercial graphite electrode. Three plateaus are observed in the quasi-equilibrium open circuit potential curves, which are indicative of a mixed phase upon lithium-ion intercalation/deintercalation. The obtained diffusion coefficients tend to increase with increasing lithium concentration and exhibit an insignificant difference between charge and discharge conditions. This study reveals that the diffusion coefficient of graphite obtained with the GITT (1 × 10^−11^ cm^2^/s to 4 × 10^−10^ cm^2^/s) is in reasonable agreement with literature values obtained from electrochemical impedance spectroscopy. The GITT is comparatively simple and direct and therefore enables systematic measurements of ion intercalation/deintercalation diffusion coefficients for secondary ion battery materials.

## 1. Introduction

Over the last three decades, lithium-ion batteries (LIBs) have been employed for diverse energy storage applications ranging from portable electronics to stationary energy storage systems [1,2]. The performance of LIBs is determined by lithium-ion diffusion in both the anode and cathode, which is furthermore coupled to electron transport [3]. For the practical use of electrodes, the lithium-ion diffusion coefficient of an electrode can be used as a performance descriptor [4,5,6,7].

It is generally recognized that graphite anodes limit the rate capability of commercial LIBs due to slow lithium-ion diffusion, allowing only a rate of up to 1C for the charging process in order to prevent lithium metal plating on the graphite surface in fast charge regimes [3]. The lithium-ion diffusion coefficient of graphite has been reported across seven orders of magnitude (from 10^−12^ to 10^−5^ cm^2^/s), as determined from electrochemical impedance spectroscopy (EIS) [8,9,10,11,12,13,14], galvanostatic intermittent titrations [15,16], and potentiostatic intermittent titrations [17,18]. EIS is a useful tool for identifying the contribution of different charge transfer and transport processes to overall electrochemical cell impedance [19,20]. EIS with subsequent equivalent circuit model fitting has been widely used to determine the diffusion coefficient of graphite [8,9,10,11,12,13,14]. However, diffusion coefficients measured by EIS are sensitive to differences in particle size and shape of the electrode materials [21], resulting in seemingly disparate diffusion coefficients in the literature. Conversely, the galvanostatic intermittent titration technique (GITT) is insensitive to particle size and electrode shape, which allows both thermodynamic and kinetic parameters to be determined, including the lithium-ion diffusion coefficient [21]. GITT measurements are composed of a series of small positive (or negative) current pulses followed by a relaxation period, where no current passes through the cell. This allows for the quasi-equilibrium open circuit voltage (open circuit voltage as a function of the state of charge) to be obtained as a function of intercalated ion concentration. Furthermore, the diffusion coefficient can be extracted by monitoring the change in potential with time under the assumption of Fick’s law. Despite there being various reports on graphite analysis using EIS and the GITT, there are few detailed studies that use the GITT to evaluate the diffusion coefficient of graphite as a function of lithium-ion concentration and potential.

In this study, we prepared a coin cell using a commercial graphite electrode sheet as the counter electrode and lithium metal as the reference electrode. The charge–discharge behavior of the coin cell was performed as a function of charge rate, confirming reversible lithium-ion intercalation/deintercalation below 0.2C. GITT experiments were performed with three different C-rates (i.e., 0.2C, 0.05C, and 0.01C), revealing that 0.05C is the optimum condition for extracting the quasi-equilibrium open circuit potential and lithium-ion diffusion coefficient of graphite. To the best of our knowledge, this is the first systematic study on the lithium-ion diffusion coefficient of graphite as a function of lithium-ion concentration and potential.

## 2. Materials and Methods

A commercial graphite electrode sheet was provided from LIBEST (Daejeon, Korea). This graphite electrode sheet was composed of 91 wt.% graphite (See the Figure S1) as the active material, 1 wt.% Super-P carbon black as a conductive additive, and 8 wt.% polyvinylidene fluoride as a binder polymer. The electrode was cast on a copper foil (10 μm thickness), dried, roll pressed, and then punched into discs 1.5 cm in diameter to obtain a mixture loading level of 8.68 mg/cm^2^ with a thickness of 100 μm (the overall thickness of the graphite electrode sheet was 110 μm). The 2032-type coin cells were then assembled with a prepared electrode disc as the cathode and a lithium metal foil as the anode using 1 M lithium hexafluorophosphate in ethylene carbonate (EC) and ethyl methyl carbonate (EMC) as the electrolyte (EC/EMC, 3:7 *w*/*w*).

**Figure 1:**
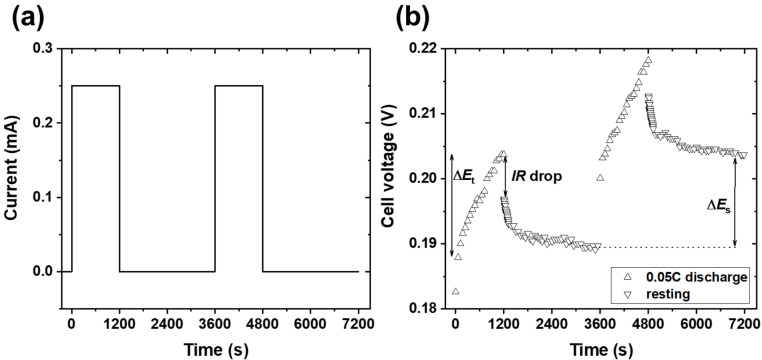
(**a**) Galvanostatic intermittent titration technique (GITT) experimental conditions during charge at 0.05C. (**b**) Corresponding variations in potential during the 0.05C charging current pulses and relaxation periods, where Δ*E_t_* is the overall change in potentials during the current pulses after subtracting the IR drop and Δ*E_s_* is the change in steady-state potential.

Electrochemical characterization of the coin cell was performed using a battery test system (Land Instruments, CT2001A). The coin cell was first tested at 27 °C in order to investigate the specific capacity and Coulombic efficiency. GITT experiments were conducted to determine the quasi-equilibrium open circuit potential (QOCP) and lithium-ion diffusion coefficients as functions of the lithium-ion concentration in graphite. For the electrochemical experiments, the upper and lower cut-off potentials were set to 1.5 and 0.06 V vs. lithium metal, respectively. For the GITT experiments, a series of current pulses were imposed on the cell for 1200 s, after which the relaxation potentials of the cell were measured for 2400 s when no current was applied. Figure 1a shows GITT experimental condition during charge at 0.05C. Figure 1b shows representative GITT curves under charging conditions of 0.05C. When a positive current pulse was applied, the potential first increased quickly—corresponding to the electrical internal resistance of the electrode—and subsequently increased slowly due to electrochemical lithium-ion deintercalation upon galvanostatic charging. After reaching a certain cell potential, due to electrical internal resistance, the potential instantaneously dropped, after which it slowly decreased until reaching equilibrium at the QOCP. When a negative current pulse was applied, the opposite held true. The diffusion coefficient (*D*) can be calculated at each step as follows:

$$D=\frac{4}{\pi \tau }{\left(\frac{nV}{S}\right)}^{2}{\left(\frac{\Delta E_{s}}{\Delta E_{t}}\right)}^{2},\;\tau <\frac{L^{2}}{D}$$

where *τ* is the duration of the current pulse (s), *n* is the number of moles (mol) of the electrode, *V* is the molar volume (cm^3^/mol) of the electrode, *L* is the thickness of the electrode, and *S* is the apparent electrode area (cm^2^) [22,23]. As shown in Figure 1b, Δ*E_t_* is the potential change for the charge/discharge current pulse, while Δ*E_s_* is the steady-state voltage change after eliminating the IR drop originating from the electrical internal resistance.

## 3. Results and Discussion

**Figure 2:**
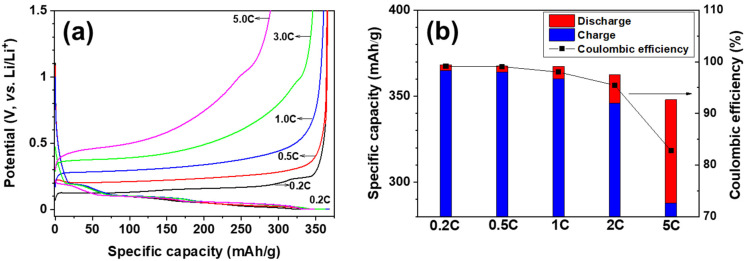
(**a**) Rate capability measured at 0.2C for discharging and at various rates for charging. (**b**) Summary of specific capacity and Coulombic efficiency.

Figure 2a shows the galvanostatic charge–discharge curves as a function of charging C-rate (0.2C, 0.5C, 1C, 2C, and 5C) when the discharging C-rate was fixed at 0.2C. At 0.2C, the highest charge capacity and Coulombic efficiency were 365 mAh/g and 99.5%, respectively (Figure 2b). Both charge capacity and Coulombic efficiency decreased with increasing C-rate due to sluggish lithium intercalation kinetics. Therefore, among the C-rates analyzed, 0.2C was the highest that could be used to perform GITT measurements to determine the diffusion coefficient for reversible lithium-ion intercalation/deintercalation into graphite, with respect to specific capacity and Coulombic efficiency. Since the GITT measurements included a series of current pulses followed by a relaxation period, the optimal C-rate for GITT measurements could be different from that used in the galvanostatic charge/discharge experiments. Therefore, three different C-rates (0.2C, 0.05C, and 0.01C) were used in the GITT measurements and were examined to extract the quasi open circuit potential and the diffusion coefficient.

**Figure 3:**
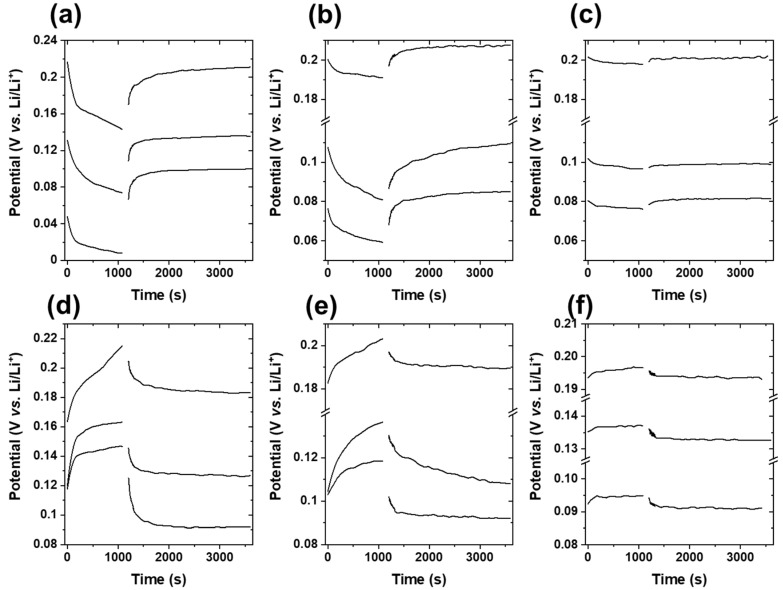
Variations in potential during current pulses for 1200 s and a relaxation period of 2400 s at the three, representative discharge (top)/charge (bottom) C-rates: (**a**,**d**) 0.2C, (**b**,**e**) 0.05C, and (**c**,**f**) 0.01C.

**Figure 4:**
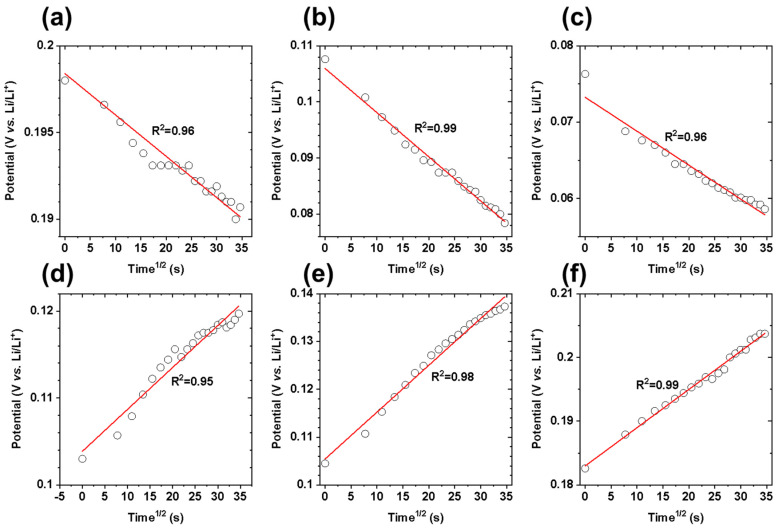
Changes in potential during the current pulses as a function of time^1/2^ during three representative discharge (top, (**a**–**c**)) and three charge (bottom, (**d**–**f**)) processes at a rate of 0.05C. The linear fitting line and R^2^ value of the linear regression are also displayed. Diffusion is rate-limiting for the electrochemical ion intercalation/deintercalation process when the potential change during the current pulse exhibits linear behavior with respect to the square root of time; this is because the diffusion coefficient determined by the GITT is based on Fick’s law.

As shown in Figure 3, GITT measurements were performed at 0.2C, 0.05C, and 0.01C charge/discharge rates. The potential change during the current pulse and relaxation process at 0.01C was insignificant compared to those obtained at 0.2C and 0.05C rates. This suggests that the duration of both the current pulse and the relaxation process must increase substantially to observe a significant potential change at 0.01C. By contrast, the GITT curves at 0.2C and 0.05C displayed a change in the potential for both the current pulse and relaxation process, which originated from lithium-ion intercalation/deintercalation and ion redistribution after intercalation in graphite, respectively. According to the GITT theory [22,23], the change in potential during the current pulse must exhibit linear behavior as a function of the square root of time. Figure 4 shows the potential change of the current pulse as a function of the square root of time at 0.05C charge/discharge rate, which exhibits linear behavior with R^2^ > 0.95. Furthermore, weak linear behavior of the potential change of the current pulse was observed at 0.2C and 0.01C charge/discharge rates (Figures S2 and S3), particularly for the charge pulse (0.61 < R^2^ < 0.98). This suggests that the optimum charge/discharge rate was 0.05C for the GITT investigation.

**Figure 5:**
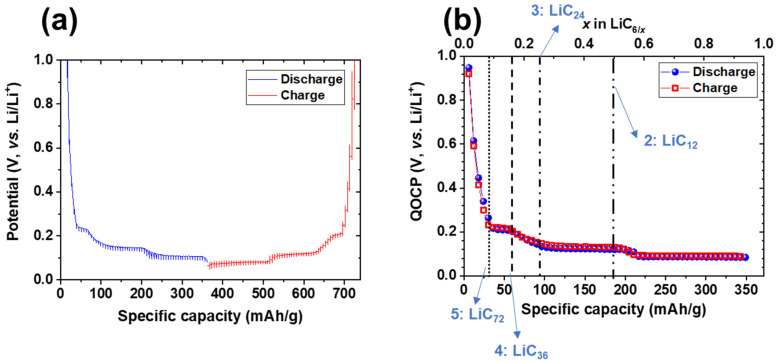
(**a**) GITT curves and (**b**) quasi-equilibrium open circuit potential (QOCP) values during charge/discharge at a rate of 0.05C.

The GITT measurement curves and QOCP values during charge/discharge at 0.05C rate are shown in Figure 5. QOCP is also plotted as a function of lithium concentration in graphite (x in LiC_6/x_), displaying three different plateaus. Low-level hysteresis was observed between charging and discharging with x < 0.08, as this low concentration of intercalated lithium resulted in random intercalation of lithium ion in graphite. Even though a large hysteresis between lithium insertion and removal on QOCP graphite was observed previously [24,25,26], no distinct hysteresis was found when x ≥ 0.08, suggesting that a rate of 0.05C is sufficiently slow to investigate lithium intercalation/deintercalation processes.

Three transients of the QOCP were observed at 1.0–0.22 V, 0.2–0.1 V, and 0.1–0.08 V, which arose from disordered lithium ions intercalated in graphite and were attributed to a significant increase in the intercalation reaction entropy and considerable change of the QOCP [24]. The plateaus are located at approximately 0.22, 0.12, and 0.08 V for both charge/discharge QOCPs. These distinct plateaus confirm that reversible lithium intercalation/deintercalation occurs because the chemical potential of the lithium ion in coexisting phases is equal with respect to the cell potential [27]. In the first plateau at ~0.22 V (0.08 ≤ x ≤ 0.17), the formation of a LiC_36_ phase (Stage 4) occurs according to LiC_72_ + Li^+^ + e^−^ ↔ 2LiC_36_. The second plateau at ~0.12 V (0.25 ≤ x ≤ 0.50) is attributed to the formation of a LiC_12_ phase (Stage 2) following LiC_24_ + Li^+^ + e^−^ ↔ 2LiC_12_. The third plateau at ~0.08 V (x ≥ 0.55) represents the reversible phase transition between LiC_12_ and LiC_6_ (Stage 1, LiC_12_ + Li^+^ + e^−^ ↔ 2LiC_6_).

**Figure 6:**
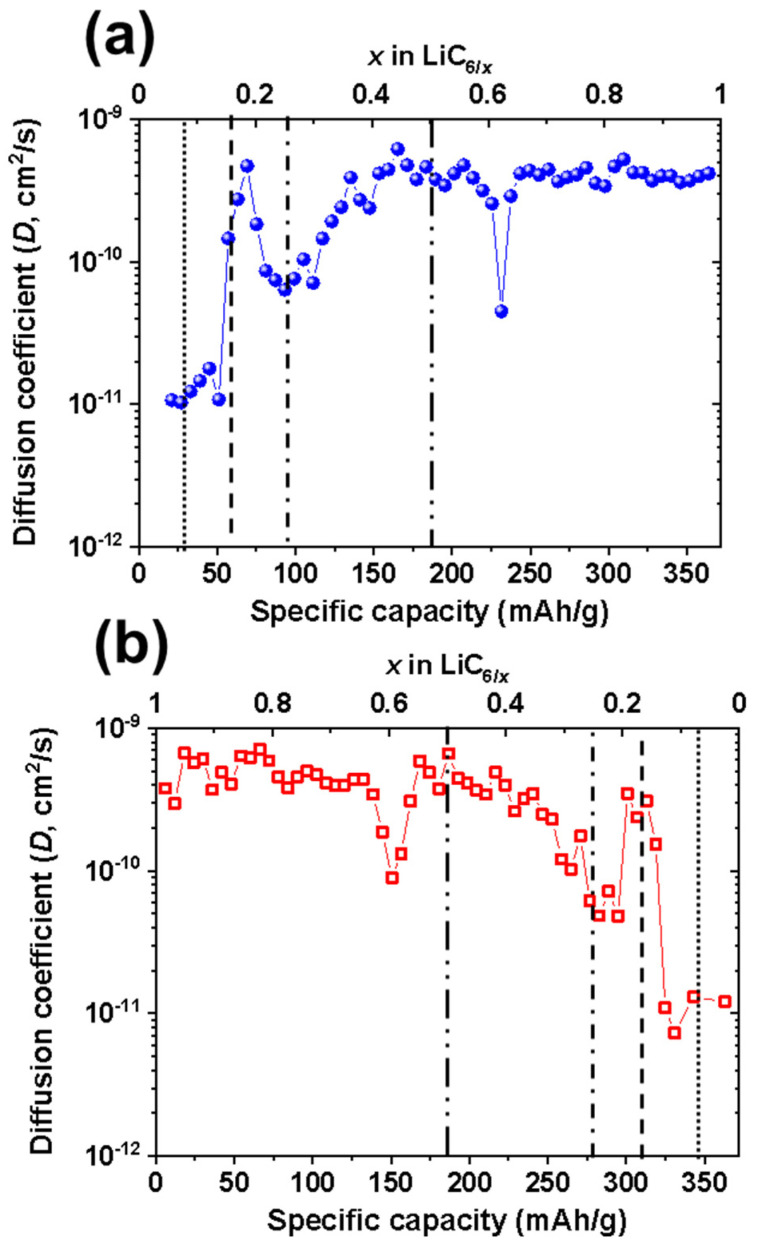
Calculated diffusion coefficient (*D*) of graphite from the GITT for discharge (top, (**a**))/charge (bottom, (**b**)) at a rate of 0.05C as a function of lithium concentration (x in LiC_6/x_).

The diffusion coefficient (*D*) of graphite during charge/discharge at a rate of 0.05C as a function of lithium concentration (x in LiC_6/x_) was investigated (Figure 6). The trends of diffusion coefficients between charge and discharge, which increase with increasing lithium concentration (x in LiC_6/x_), are nearly identical. When x < 0.16, the diffusion coefficient is ~10^−11^ cm^2^/s; because the d-spacing of graphite undergoes an insignificant change from 3.36 Å to 3.39 Å upon lithium-ion intercalation [28], a high overpotential for both the insertion and removal of lithium ions occurs. In situ synchrotron X-ray diffraction results confirmed that the symmetry of the (0 0 2) peak of graphite exhibited higher symmetry upon lithium-ion intercalation due to the strong repulsion between intercalated lithium ions [25,27,28], resulting in an increased diffusion coefficient. In the range of 0.16 ≤ x ≤ 0.2, the diffusion coefficient substantially increased approximately 10 fold to ~2 × 10^−10^ cm^2^/s, since the d-spacing of graphite increased significantly from 3.39 Å to 3.49 Å, thus providing sufficient space for lithium-ion diffusion in conjunction with the formation of the LiC_36_ phase [28]. The diffusion coefficient decreased slightly to approximately 8 × 10^−11^ cm^2^/s in the 0.2 ≤ x ≤ 0.25 range due to formation of LiC_24_, which is in good agreement with previous X-ray diffraction studies [25,27]; lithium ions in LiC_24_ are intercalated in a more compact manner than in the LiC_36_ phase. When 0.25 ≤ x ≤ 0.5, the diffusion coefficient was found to increase again to ~2 × 10^−10^ cm^2^/s due to the phase transition from LiC_24_ to LiC_12_ [29]. During this transition, lithium ions were sufficiently intercalated into graphite and available for diffusion from the edge to the basal plane, which is in good agreement with the high entropy associated with the charge/discharge process [24,25]. At x ≥ 0.5, the diffusion coefficient remained at approximately 10^−10^ cm^2^/s but exhibited a sharp drop for both charge/discharge curves at x = 0.6. This sudden decrease at x = 0.6 is associated with the formation of a superdense LiC_12_ intermediate phases, such as Li_7_C_24_ or Li_11_C_24_ [25], acting as nucleation sites for the formation of LiC_6_ as observed in in situ X-ray diffraction. This is because the presence of intermediate phases leads to a lithium concentration gradient between the surface and interior of graphite particles, which hinders lithium-ion diffusion.

**Figure 7:**
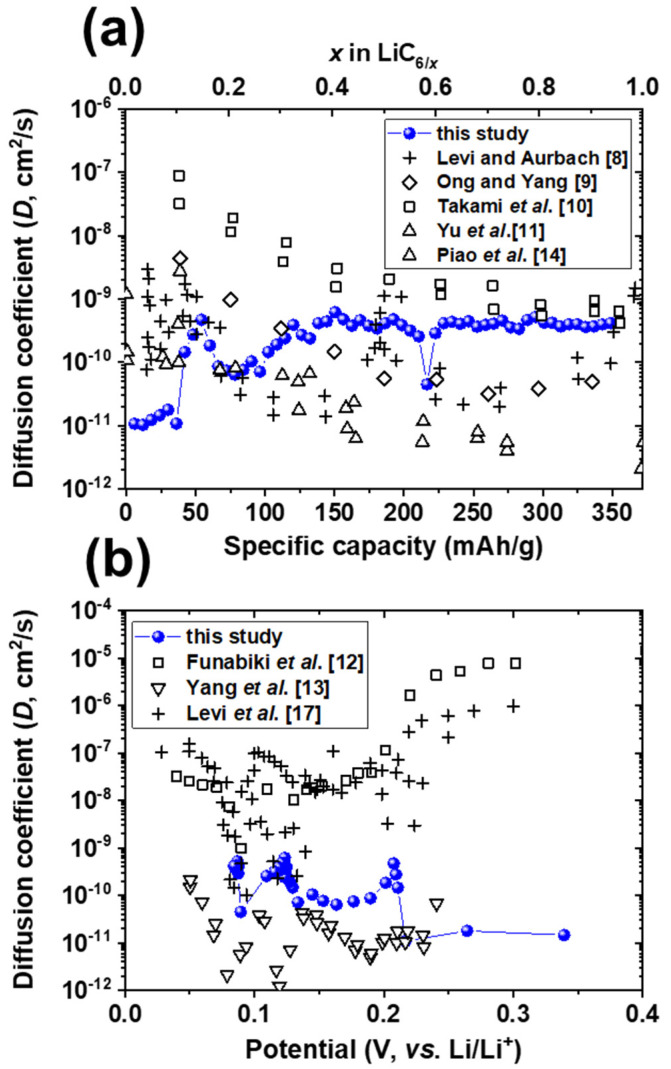
Survey of experimental and literature diffusion coefficient (*D*) values for lithium-ion intercalation in graphite as functions of (**a**) specific capacity (x in LiC_6/x_) and (**b**) potential (vs. Li^+^/Li).

The diffusion coefficients of lithium-ion intercalation in graphite as a function of specific capacity (x in LiC_6/x_) and potential (vs. Li^+^/Li) from different EIS studies are compared in Figure 7. Only a single diffusion coefficient value of 4 × 10^−8^ cm^2^/s was previously reported using the GITT with a charge current of 0.2 mA [15], which is similar to this study. Recently, diffusion coefficient values of graphite in the range of 1 × 10^−13^ to 2 × 10^−11^ cm^2^/s determined from the GITT have been reported using relatively rapid C-rates from 0.2C to 5C [16]. However, lithium-ion diffusion coefficients of graphite as functions of specific capacity and potential have not yet been reported using the GITT. In fact, most reported lithium-ion diffusion coefficients as functions of specific capacity and potential are obtained from EIS [8,9,10,11,12,13,14] and potentiostatic intermittent titration technique measurements [17]. Even though discrepancies between lithium-ion diffusion coefficients could arise from differences in the structure of graphite particles, electrode sheet composition, and analysis techniques, the lithium-ion diffusion coefficient obtained from this study is in reasonable agreement with the results from Levi and Aurbach [8] and Ong and Yang [9] as a function of x in LiC_6/x_, and with those of Yang et al. [13] as a function of potential. It has been demonstrated that the GITT enables the direct and reliable determination of the lithium-ion diffusion coefficient of graphite when the lithium-ion intercalation/deintercalation reaction occurs reversibly.

## 4. Conclusions

We investigated the quasi-equilibrium open circuit potential and lithium-ion diffusion coefficient of a graphite electrode by employing the galvanostatic intermittent titration technique as a function of intercalated lithium-ion concentration (x in LiC_6/x_) and potential. We determined that the optimum charge/discharge rate was 0.05C for investigation of the quasi-equilibrium open circuit potential and lithium-ion diffusion coefficient of graphite. Furthermore, three distinct plateaus were identified from the quasi-equilibrium open circuit potential curves at approximately 0.22, 0.12, and 0.08 V; the first plateau at ~0.22 V (0.08 ≤ x ≤ 0.17) corresponds to the phase transition from LiC_72_ to LiC_36_, the second plateau at ~0.12 V (0.25 ≤ x ≤ 0.50) is related to the phase transition from LiC_24_ to LiC_12_, and the third plateau at ~0.08 V (x ≥ 0.55) corresponds to the phase transition from LiC_12_ to LiC_6_. Finally, the calculated diffusion coefficient values from the galvanostatic intermittent titration technique were in the range of 1 × 10^−11^ cm^2^/s to 4 × 10^−10^ cm^2^/s, showing fair agreement with previous results obtained using electrochemical impedance spectroscopy. We believe that once reversible electrochemical intercalation/deintercalation conditions are established, the galvanostatic intermittent titration technique can be universally applied as a powerful tool to determine the ion diffusion coefficients of graphite-based electrodes.

## Data Availability

All data included in this study are available upon request by contact with the corresponding author.

## Supplementary Materials

The following are available online at https://www.mdpi.com/article/10.3390/ma14164683/s1: Figure S1: X-ray diffraction pattern and secondary electron microscopy image of graphite powder; Figure S2: Change in potential during the current pulse with time^1/2^, and the linear fitting line, and R^2^ of the linear regression during discharge (top)/charge (bottom) at a rate of 0.2C; and Figure S3: Change in potential during the current pulse with time^1/2^, and the linear fitting line, and R^2^ of the linear regression during discharge (top)/charge (bottom) at a rate of 0.01C.
