# Supplementary material for: Investigation of Lithium Ion Diffusion of Graphite Anode by the Galvanostatic Intermittent Titration Technique
Source: Materials (Basel). 2021 Aug 19;14(16):4683. doi: 10.3390/ma14164683 (PMC8397968; doi:10.3390/ma14164683)
Supplement: Supplementary file 1 [file materials-14-04683-s001.zip › materials-1296012-SM.pdf]

Supporting Information

Investigation of Lithium Ion Diffusion of Graphite Anode by the Galvanostatic Intermittent Titration Technique

Jong Hyun Park [1], Hana Yoon [1], Younghyun Cho [2] and Chung-Yul Yoo [3,*]

[1] Energy Conversion & Storage Materials Research Laboratory, Korea Institute of Energy Research, 152 Gajeong-ro, Yuseong-gu, Daejeon 34129, Korea; whdgus615@gmail.com (J.H.P.); hanayoon@kier.re.kr (H.Y.)

[2] Department of Energy Systems Engineering, Soonchunhyang University, Asan 31538, Korea; yhcho@sch.ac.kr

[3] Department of Chemistry, Mokpo National University, Muan-gun 58554, Korea

* Correspondence: chungyulyoo@mokpo.ac.kr; Tel.: +82-61-450-2335; Fax: +82-50-4766-5737

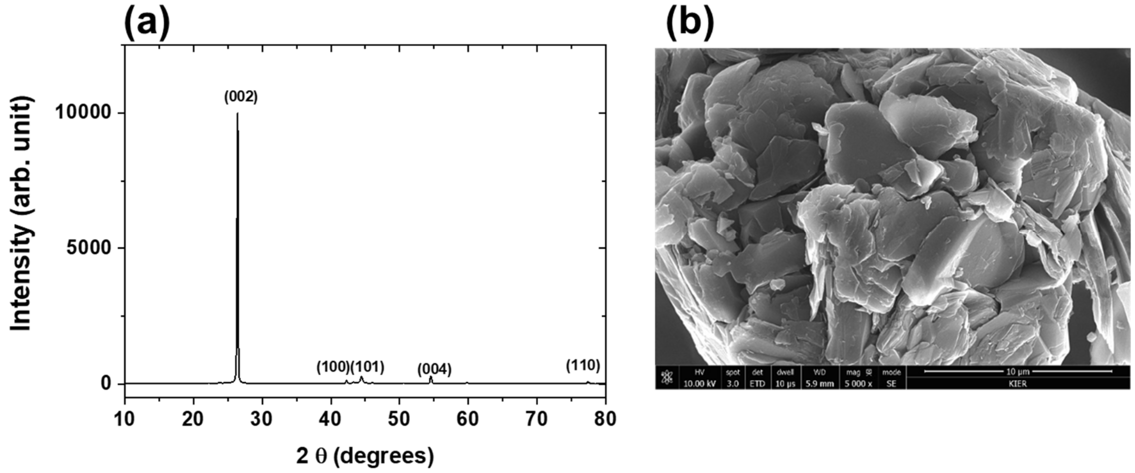

Figure S1 (a) X-ray diffraction pattern and (b) secondary electron microscopy image of graphite powder from LIBEST.

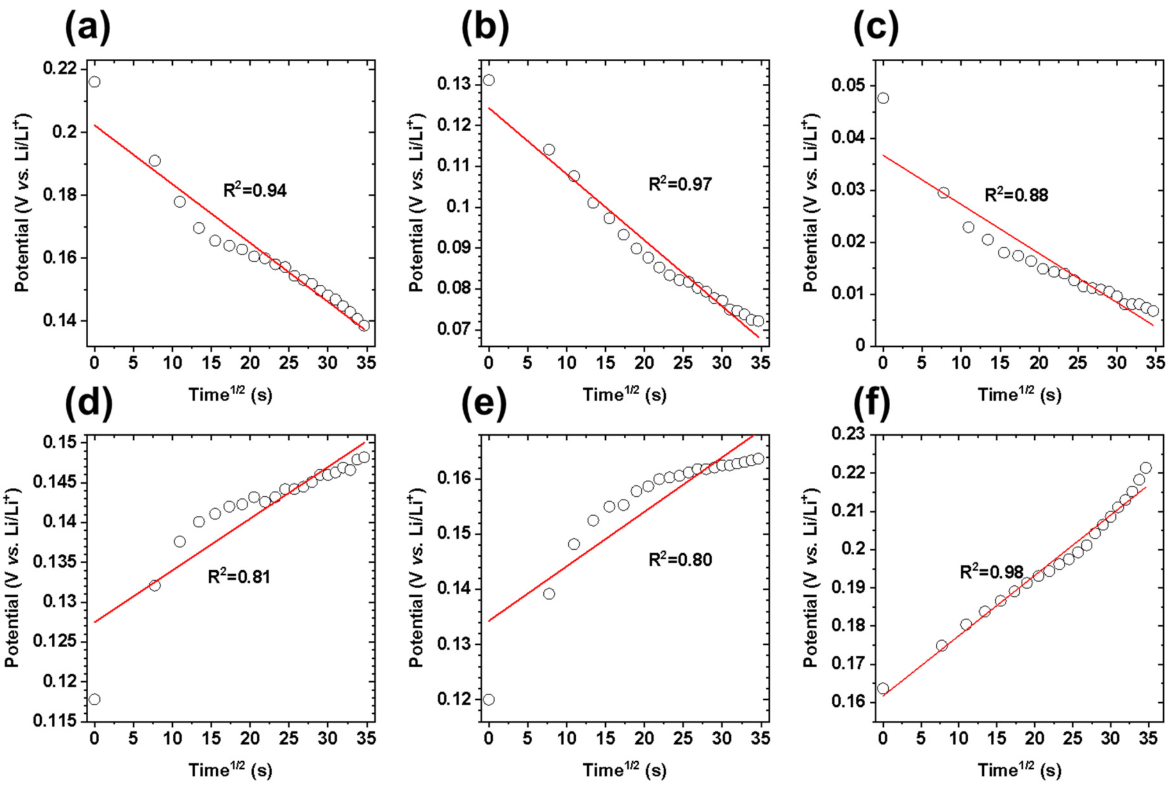

Figure S2 Change in potential during the current pulse with time$^{1/2}$, and the linear fitting line, and R^2 of the linear regression during discharge(top)/charge(bottom) at a rate of 0.2C. The linear fitting line and R^2 value of the linear regression are also displayed. Diffusion is rate-limiting for the electrochemical ion intercalation/deintercalation process when the potential change during the current pulse exhibits linear behavior with respect to the square root of time; this is because the diffusion coefficient determined by the GITT is based on Fick's law.

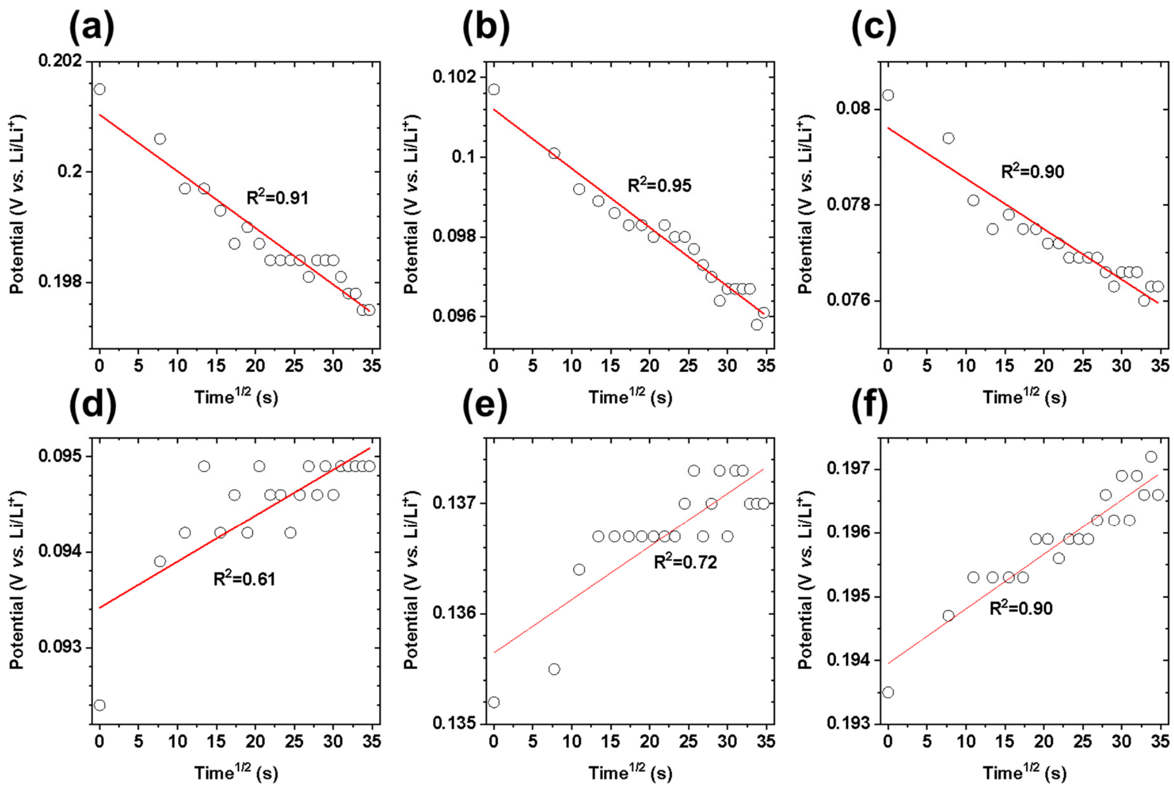

Figure S3 Change in potential during the current pulse with time$^{1/2}$, and the linear fitting line, and R^2 of the linear regression during discharge(top)/charge(bottom) at a rate of 0.01C. The linear fitting line and R^2 value of the linear regression are also displayed. Diffusion is rate-limiting for the electrochemical ion intercalation/deintercalation process when the potential change during the current pulse exhibits linear behavior with respect to the square root of time; this is because the diffusion coefficient determined by the GITT is based on Fick's law.
